# The Horace Wells Anniversary Celebration

**Published:** 1894-11

**Authors:** 


### THE HORACE WELLS ANNIVERSARY CELEBRATION.

Upon another page will be found the circular letter of the committee appointed by the American Dental Association to celebrate the semi-centennial of the discovery of anæsthesia.

The committee have perfected the arrangements, which include two papers to be read, one upon the "History of Anæsthesia," by Professor Fillebrown, of Boston, and one on the "Benefits of Anæsthesia to Mankind," by Professor Garretson, of Philadelphia, to be followed in the evening by a banquet, at which it is presumed many invited guests will be present from the medical profession. The cost of this has been placed at six dollars.

The importance of this occasion cannot be overstated. It means more than a celebration of a mere event, for it emphasizes the fact that at this period fifty years ago was discovered the means to relieve humanity from suffering during prolonged operations, and has, in the advance of years, enabled surgeons to perform operations undreamt of in the period prior to its introduction. More than this, it will positively settle the date and the honor of this important discovery. Fifty years of contention have served to free this disputed question of all difficulties, and the time is now an appropriate one to celebrate the event.

To dentistry this marks an epoch, and should be regarded as such by every man engaged in the work. It is therefore hoped that as many as possible will find it convenient, as well as a duty, to be present to make the occasion an imposing one.

The criticism has been made that something more substantial than a meeting, addresses, and a banquet should mark this period. In this we heartily concur; but it is understood that at the meeting measures will be taken for a more permanent recognition of Dr. Wells's work. This may possibly assume the form of a monument, to be erected in some one of the large cities. It is too early to consider this, but if this be decided upon as the most appropriate form for a memorial to take, Washington should be selected, as it has claims above all other places.

The privilege of contributing to this fund should not be confined to the dentists of the United States, but contributions should be solicited from the profession of the world. The honor is theirs as much as ours, and in this work there should be no national lines.

There should be no question as to the possibility of raising sufficient funds to build a monument worthy the man and the great service he performed for the alleviation of suffering, and we believe it could speedily be accomplished.

